# Structural design of the echinoid’s trabecular system

**DOI:** 10.1371/journal.pone.0204432

**Published:** 2018-09-27

**Authors:** Tobias B. Grun, James H. Nebelsick

**Affiliations:** Department of Geosciences, University of Tübingen, Tübingen, Germany; University of Memphis, UNITED STATES

## Abstract

The multi-plated skeleton of echinoids is made of the stereom, a light-weight construction which resembles a micro-beam framework. Although the two-dimensional design of the stereom has been studied, its spatial architecture is only little known. It is, however, imperative to understand the spatial architecture of the trabecular system in order to interpret its structural principles of this load-bearing construction. The echinoid’s trabecular system is thus analyzed in-depth with respect to eight topological descriptors. The echinoid’s plates are divided into two regions, the center of which consists of an unordered stereom, and the margin which consists of an ordered stereom. The eight trabecular descriptors indicate that the basal topology of the two plate regions are similar. The trabecular system predominantly consists of short and stocky trabeculae that show little tortuosity. The majority of trabeculae intersect in a 3N configuration, where three trabeculae intersect in one common node. Trabeculae in the 3N configuration intersect in an angle of around 120° resulting in a planar and triangular motif. These planar elements, when arranged in an angular off-set, can resist multi-dimensional loads. Results also show that the trabecular orientation perpendicular to the plate’s surface is at an angle of 60°. The trabecular orientation in the plate’s horizontal plane is directional. Both trabecular orientations reflect a construction which is capable of resisting applied loads and can distribute these loads over the entire skeleton. The spatial architecture of the echinoid’s trabecular system is thus considered to be a performative light-weight and load-bearing system.

## Introduction

**Fig 1:**
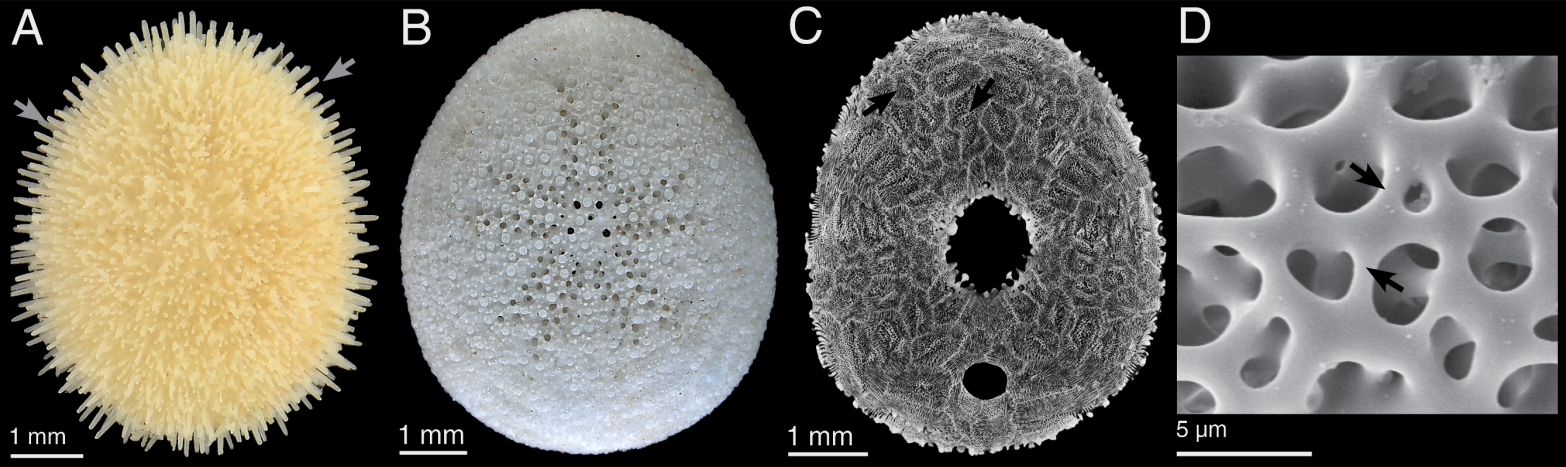
The echinoid skeleton is hierarchically organized. (A) *Echinocyamus pusillus* with spines (arrows). (B) Denuded skeleton. (C) Micro-CT section showing individual plates (arrows). (D) Micrograph of the stereom network and trabeculae (arrows).

The echinoid’s skeleton is a hierarchical multi-element construction (Fig 1), consisting of numerous individual skeletal parts, including the plates from which the test (shell) is formed, spines, skeletal discs of the tube-feet, and skeletal elements of the pedicellariae [1, 2] These minute skeletal elements are constructed of the stereom, which resembles a micro-beam system (Fig 1D). The stereom of the echinoid spines has been investigated for its structural design [3–6] and mechanical performance [6–8] using both 2-dimensional (2d) images and 3-dimensional (3d) models. The stereom of the plates was likewise examined by 2d imaging [3, 9, 10], but 3d analyses are still lacking. The 3d analyses of the echinoid’s trabecular system are, however, of highest interest, as they allow for a detailed understanding and interpretation of the structural context and mechanical behavior, which cannot be obtained from 2d analytics.

Echinoids of the order Clypeasteroida have become the focus of structural research as their often flattened tests demonstrate a high strength and robustness [11, 12–16]. The stable nature of these tests is not only reflected in the abundances of complete test or their stable fragments in recent environments [15, 17–19], but also in the rich fossil record [14, 15, 20–24]. The structural integrity of the clypeasteroid tests is a result of the plate arrangement [16], plate interlocking, and internal support systems [13, 15, 16, 18, 25]. The tests of clypeasteroid echinoids are thus of interest of biomimetic research, which aims to identify structurally performative constructions that potentially can be transferred into architectural and engineering disciplines. The mechanisms and principles of such structures are used to improve technical multi-element constructions [14]. The structural design of the echinoid’s trabecular system is here analyzed for the first time in-depth using x-ray micro-computed tomography and is interpreted in the context of engineering concepts.

### Trabecular descriptors

Trabecular descriptors are morphological parameters describing the spatial architecture of a structure [26]. These parameters can thereby yield information on the structural mechanics of the trabecular structures. Eight trabecular descriptors are described that possess the potential for structural mechanic interpretations of the echinoid’s trabecular system: (1) trabecular length, including the curved length and the chord length, (2) trabecular tortuosity, (3) trabecular radius, (4) trabecular slenderness ratio, (5) inter-trabecular angle, (6) node configuration, (7) theta orientation, and (8) phi orientation.

### Trabecular length, tortuosity, radius and slenderness ratio

These descriptors are closely associated to one another. Following structural mechanic principles, a slender trabecula is more subject to buckling than a stocky trabecula at a given length demonstrated by Eq (1), where the radius *r* is to the power of four and hence can significantly influence flexural stiffness *EI*, which is the product of the material depended Young’s modulus *E* and the second moment of area *I*.

$$I=\frac{\pi r^{4}}{4}$$ (1)

*I* = second moment of area, *r* = trabecular radius. Any increase in *r* will increase *I*, and hence the flexural stiffness *EI*, which prevents the trabeculae from buckling, as expressed in Eq (2).

$$F_{E}=\frac{n\pi^{2}EI}{L^{2}}$$ (2)

*F*_*E*_ = critical Euler buckling force, *n* = coefficient for segments with two fixed ends = 4 [27–29], *E* = Young’s Modulus, *EI* = second moment of area, *L* = trabecular length.

A related parameter is the slenderness ratio, which is defined as the ratio between the trabecular length and the trabecular radius of gyration.

$$R_{S}=\frac{L}{g_{r}}$$ (3)

*R*_*S*_ = slenderness ratio, *L* = trabecular length, *g*_*r*_ = trabecular radius of gyration, where

$$g_{r}=\frac{d_{t}}{4}$$ (4)

*d*_*t*_ = trabecular diameter. Any increase in trabecular length at a given trabecular radius *r* decreases the critical Euler buckling force, which is force at which structural failure is expected [28]: loadings on an ideal beam in longitudinal axis exclusively induces compression stress resultants. Any deviation of an ideal beam results in bending moments along the longitudinal axis of a beam, which can be countered as long as the inner bending force is equal or larger than the outer bending force. The beam can return to its initial position after the load is removed. Structural failure occurs, however, when the outer bending force exceeds the inner bending force [28]. This is usually the case when the critical Euler buckling value is reached.

### Inter-trabecular angle and node configuration

The terminology and usage of the inter-trabecular angle (ITA) was introduced by Reznikov et al. [26] for the cancellous bones of vertebrates. This topological parameter describes the trabecular architecture irrespective of trabecular length and thickness and was initially developed to correlate the angle between collagenous fibers on the micrometer level, and the angle between trabeculae at the millimeter scale [26]. The number of trabeculae intersecting in one common node (node configuration) has been interpreted with respect to its structural relevance and its conservation potential within vertebrates. In cases where three trabeculae intersect in a common node, the ideal value for identical angles is 120 degrees. This configuration eventually results in a planar triangle. In cases where four trabecular intersect in a common node, the ideal angle is 109.5 degrees resulting in a tetrahedral trabecular configuration. In nodes where more than 4 trabeculae intersect in a single node, the angular configuration and spatial appearance is more complex [26]. In vertebrate bone, it was shown that the calculated ITAs followed the *a priori* determined ideal angles [26].

### Trabecular orientations

The trabecular orientation describes the direction of a trabecula within the plate, which provides direct information of how the trabeculae are distributed in a plate or spine. The orientation of the trabeculae has been attributed to the structural functionality [10], where a uniformly distributed orientation enables the mesh-work to absorb multi-directional stress, while unbalanced distributions are indicative for directional stress regimes.

Theta orientation is the orientation of a trabecula perpendicular to the plate’s surface. This descriptor indicates the ability of the stereom to deal with vertical loads. Phi orientation describes the orientation of a trabecula in the plate’s horizontal plane. These descriptor indicates the course of stress within a plate. The loads are thereby randomly distributed within a plate when the phi orientation is approximately uniform, or the loads can be directional distributed when the distribution is unbalanced.

## Material and methods

### Material

Denuded skeletons of *Echinocyamus pusillus* were collected in summer 2010 around Giglio, Tuscany, Italy by SCUBA. From 1080 samples, one pristinely preserved skeleton [GPIT/EC/00740:gg-al-1.73] [30] was chosen for x-ray micro-computed tomography scanning. Samples are stored under repository GPIT/EC/00740 at the University of Tübingen, Germany.

### Methods

#### Computed tomography

The x-ray micro-computed tomography (μCT) scan was performed using a Phoenix Nanotom 180nF (General Electric Company Corporation, Boston, MA, USA). The specimen was scanned to a resolution of 3 μm per voxel, images were recorded to an 8-bit gray scale system with 256 levels. The specimen was affixed to the sample-tray on a wax-base with the longitudinal axis parallel to the detector plane. The scan was conducted with the parameters: voltage = 80 kV, power = 180 μA, exposure time = 800 ms, projections = 2000. Prior to scanning, the sample was cleaned for 31 min in a Bandelin DT106 (Bandelin Electronic, Berlin, Germany) ultrasonic bath and was then air-dried. Data are accessible at the dryad online repository [30].

#### Computed tomography data processing

**Fig 2:**
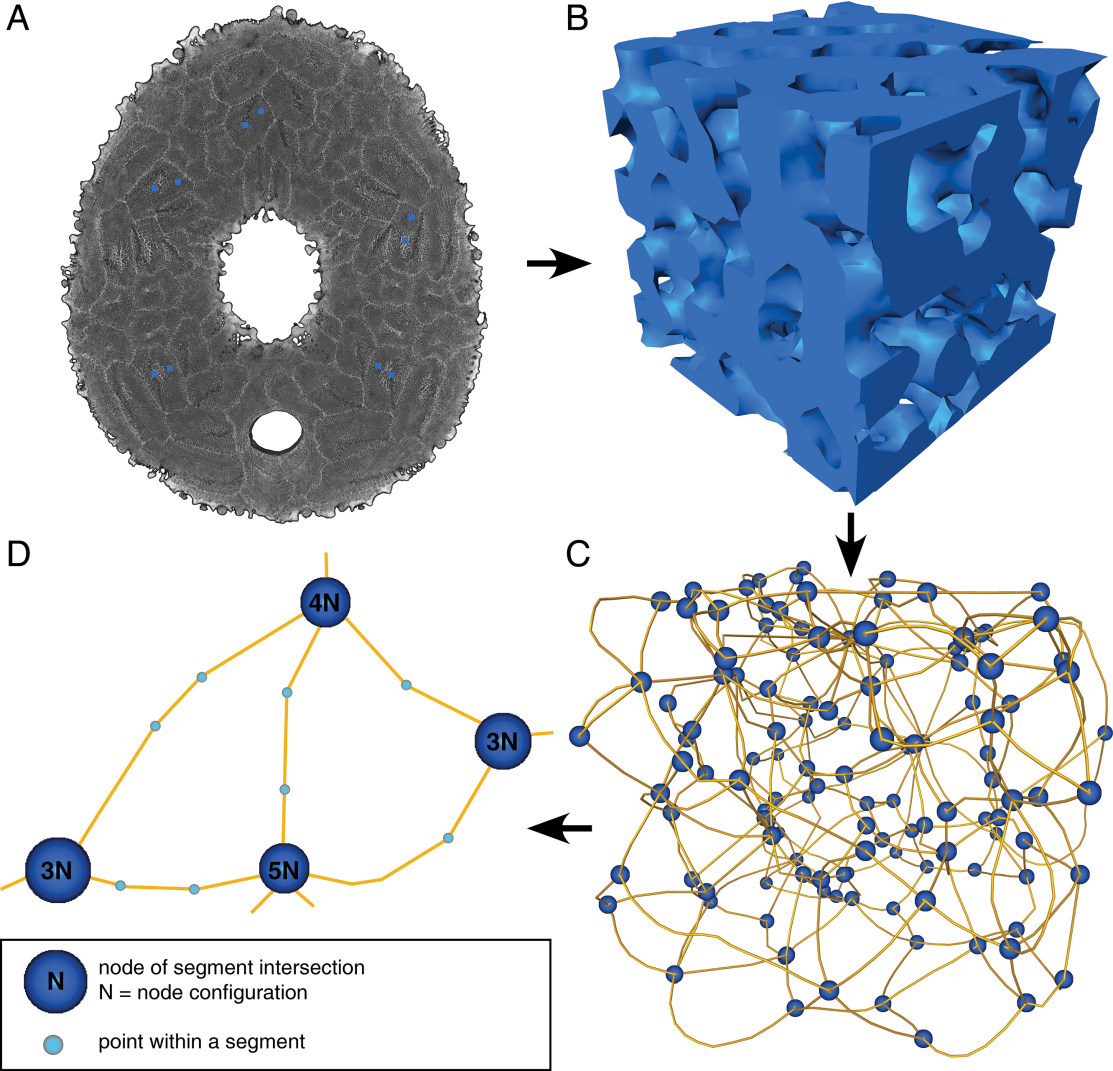
Data generation. (A) Micro-CT section of *Echinocyamus pusillus*. Blue squares indicate the areas of data origin. (B) 3d reconstruction of a subvolume of the stereom. (C) Network scheme of the stereom. Blue spheres indicate nodes; yellow lines represent trabeculae. (D) Close-up of the trabecular network. The node configuration indicates how many trabeculae intersect in a common node. The course of trabeculae is described by light-blue spheres.

Data are rendered and analyzed using Avizo in version 9.4 (Thermo Fisher Scientific, Waltham, MA, USA). Two subvolumes with an edge length of 90 μm were extracted from five plates (Fig 2A), one at the plate’s center and one at the plate’s margin. A de-noising filter was applied to the subvolumes enhancing the contrast between material (stereom) and the surrounding non-material matrix (air) (filter: delineate, interpretation = 3d, neighborhood = 26, size = 3 px). Subvolumes were binarized (Fig 2B) using the interactive thresholding algorithm (intensity range = 76–172). Areas that are not connected to the stereom were removed using the remove small spots (interpretation = 3d, size = 1000 px) function.

The subvolumes are skeletonized (Fig 2C and 2D) using the auto skeleton command (smooth = 0, attach to data = 0.5, number of iteration = 1000). Euclidean point coordinates that describe the segments (trabeculae) and nodes (intersection of trabeculae) of the stereom, as well as the trabecular mean radius and the tortuosity (curvature) of the segments were exported from Avizo using the spatial graph statistics function. The coordinates are analyzed in the R software environment in version 3.2.2 [31]. Duplicated segments or nodes, that where generated in Avizo during the automatized skeletonization process were identified and removed for the analyses.

#### Length and tortuosity

**Fig 3:**
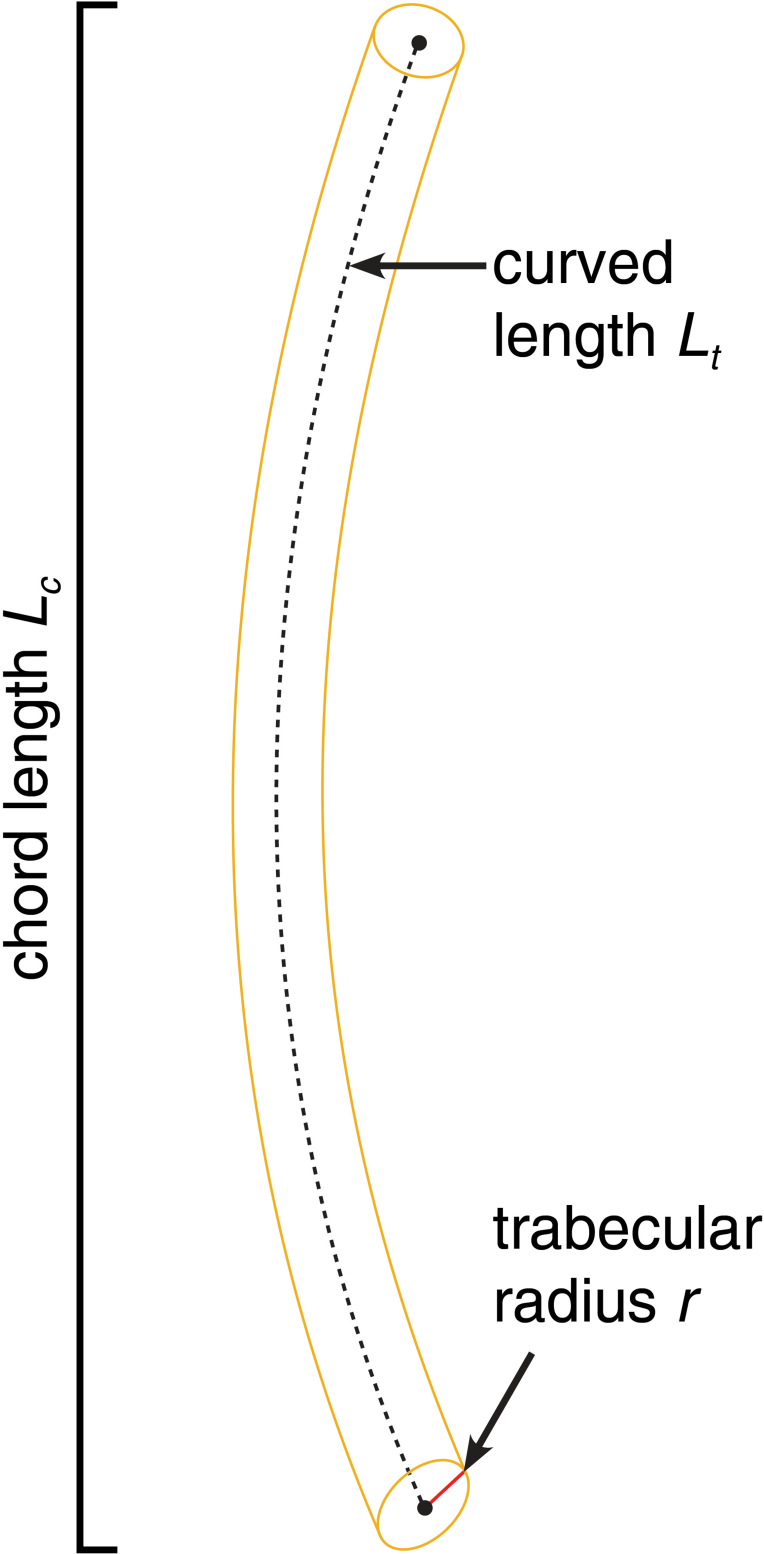
Trabecular descriptors. The trabecular length can be measured by its chord length (*L*_*c*_) which is defined by the maximum extension of a trabecula, and its curved length (*L*_*t*_) which describes the length of a trabecula following its center line. The trabecular radius is given by *r*.

The trabecular length is measured for two parameters (Fig 3). The chord length l_c_ is described by a straight line between two nodes. The curved length l_t_ represents the true and curved course of a segment. The tortuosity is the ratio between curved length and the chord length, defined by Eq (5) and describes the extent of curvature of a segment.

$$\tau =\frac{l_{t}}{l_{c}}$$ (5)

A tortuosity of τ = 1 indicates that the chord length of a segment is in equal length to the curved length, a τ > 1 indicates that a trabecula is longer than the direct connection between nodes, and thus curved. The tortuosity parameters are used to describe the course of the segments.

#### Radius and slenderness ratio

Radii are obtained from the Avizo spatial graphs statistics. The radius of a trabecula is defined as the mean value of all point radii that describe a segment, whereas the node radius is respectively the radius of a single point in which trabeculae intersect (Fig 2D). Segment and node radii are compared by a non-parametric Wilcoxon rank-sum test. Additionally, node radii and segment radii are compared between the plate’s center and margin areas. Results are reported as the median ± median absolute deviation (mad). Results are discussed with respect to their mechanical effects. Wilcoxon rank sum tests were applied to subsamples of the data so that differences in sample size and large samples do not bias the statistical test. Data are subsampled to N = 300 for each compared group, the statistical analyses ran in 10000 iterations. The mean p-value is reported for evaluation. The slenderness ratio is determined for both the center and margin regions of the plate and compared to one another using a Wilcoxon rank sum test where data are subsampled to N = 300 per group and ran for 10000 iterations.

#### Inter-trabecular angle and node configuration

The inter-trabecular angle ITA [26, 32] is the angle between any of two intersecting segments in a common node. Angle calculation is performed in two-step process, where in a first step, the position vectors of a segment are used to describe the direction vector of a segment, and in a second step, the angle between two direction vectors segments is calculated using Eq (6).

$$ITA=\cos\Theta =\frac{\vec{a}∙\vec{b}}{\left\|\vec{a}\|∙\|\vec{b}\right\|}$$ (6)

$\vec{a}$ = direction vector of a segment, $\vec{b}$ = direction vector of an $\vec{a}$ intersecting segment. The obtained ITA are averaged (mean ± standard deviation) for each node and correlated to the number of segments per node. The number of segments that intersect in one node is calculated to determine the most abundant intersections-per-node combination. The number of intersections per node follow Eq (7).

$$\sum_{i=1}^{n-1}n_{i}=\frac{n(n-1)}{2}$$ (7)

*n* = number of segments per node, *i* = index. ITA of the plate’s center and the plate’s margin are compared by a Wilcoxon rank sum test.

#### Trabeculae orientation

**Fig 4:**
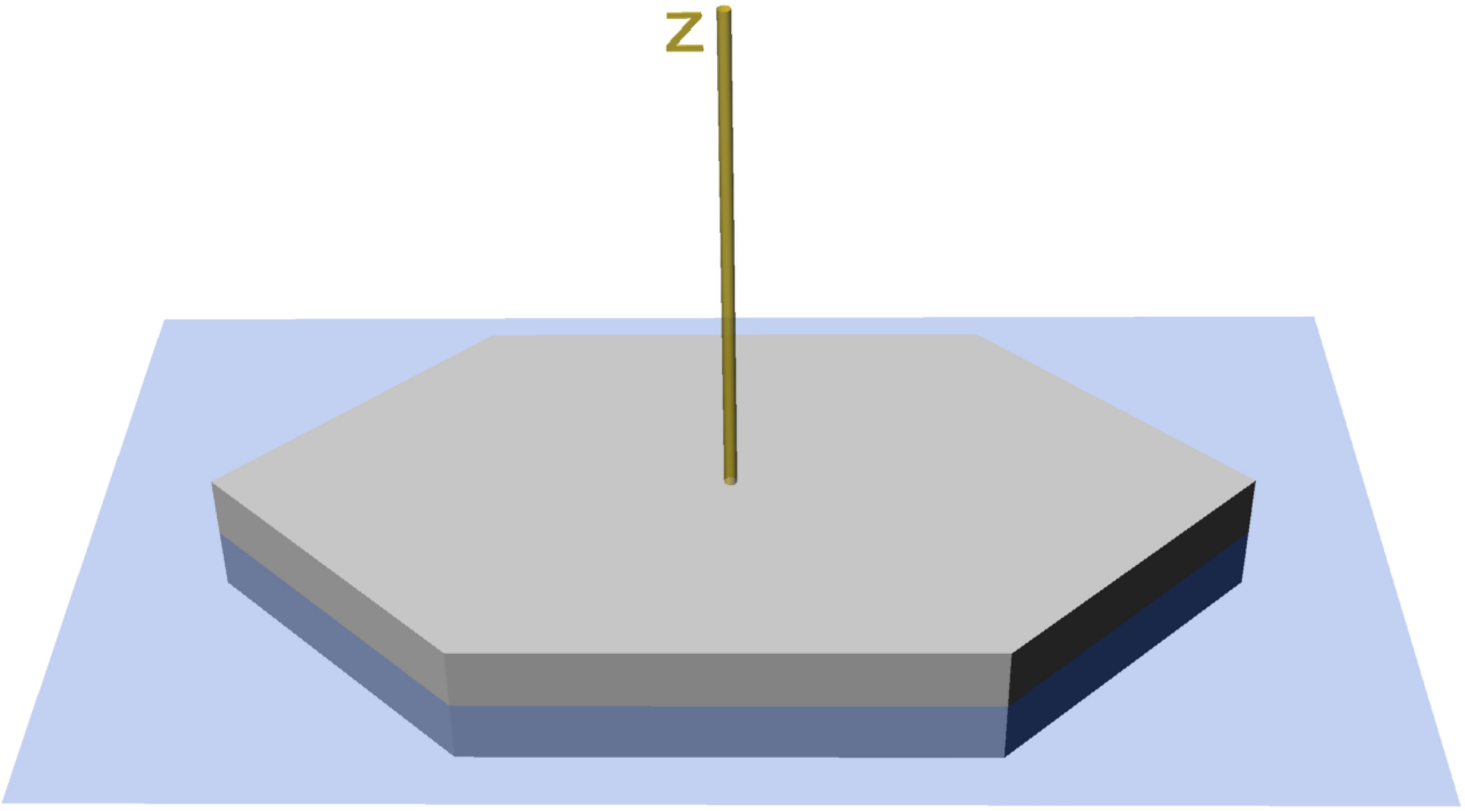
Definition of the theta and phi. Horizontal plane (blue) and the z-axis (yellow) of a plate model.

The segment orientation perpendicular to the plate’s surface is described by theta (θ), that is formed by the trabecula and the z-axis (Fig 4). Theta can range between 0 and 90 degrees, where θ = 0 degree is a trabecula perpendicular to the plate’s surface and θ = 90 degree is a trabecula in x-y direction (horizontal to the plate’s surface. The segment orientation in x-y plane is described by phi (φ), which lies in the plate’s horizontal plane (Fig 4) and can revolve from 0 to 360 degrees around the z-axis. The angles theta and phi are obtained from Avizo using the spatial graph statistics function. Their distribution provides information about the load-transfer direction. The distribution of theta is analyzed for normality using Shapiro-Wilk test for normality in R [31]. The skewness and kurtosis of the distributions is calculated by the moments-package [33] in R. The distribution of phi is analyzed for uniformity using a χ^2^ test. Visualization of the phi distribution is performed by rose diagrams using the spatstat package [34] in R. The rose diagrams have no specific orientation in relation to the skeleton, but allow an assessment of uniform or directional distributed trabeculae. The trabecular orientation is compared between the plate’s center and the plate’s margin using a Wilcoxon rank sum test.

#### Rendering

Surface models of the stereom from the plate’s center and the plate’s margin are visualized in Avizo (options: compactify = unchecked, edge length = 0.2; settings: adjust coordinates = checked; smoothening: smoothening = constrained smoothening, smoothening extend = 1) with the surface parameters (draw style = transparent; more options = specular, fancy alpha, sorting, both faces, direct normal; base trans = 0 (for non-transparent stereom view) base trans = 0.6 (for transparent stereom view)). Skeletonization results are visualized as a tube network including thickness parameters (node scale = constant, node scale factor = 1.24905, node coloring = constant, segment styles = tubes, segment coloring = thickness, segment colormap = physics, colormap range = -1.36986–3.9, tube scale factor = 0.152058).

#### Figure processing

The plate showing the horizontal plane and the z-axis was rendered in the 3d design software Rhinoceros 5 (McNeel, Seattle, WA, USA). Images were adjusted for brightness, contrast and color in Adobe Photoshop CC 2018 (Adobe Systems, San José, CA, USA). Line drawings were generated in Adobe Illustrator CC 2018 (Adobe Systems, San José, CA, USA), and final figure layout was processed in Adobe InDesign CC 2018 (Adobe Systems, San José, CA, USA).

## Results

### Length, tortuosity, radius and slenderness ratio

#### Plate center

**Fig 5:**
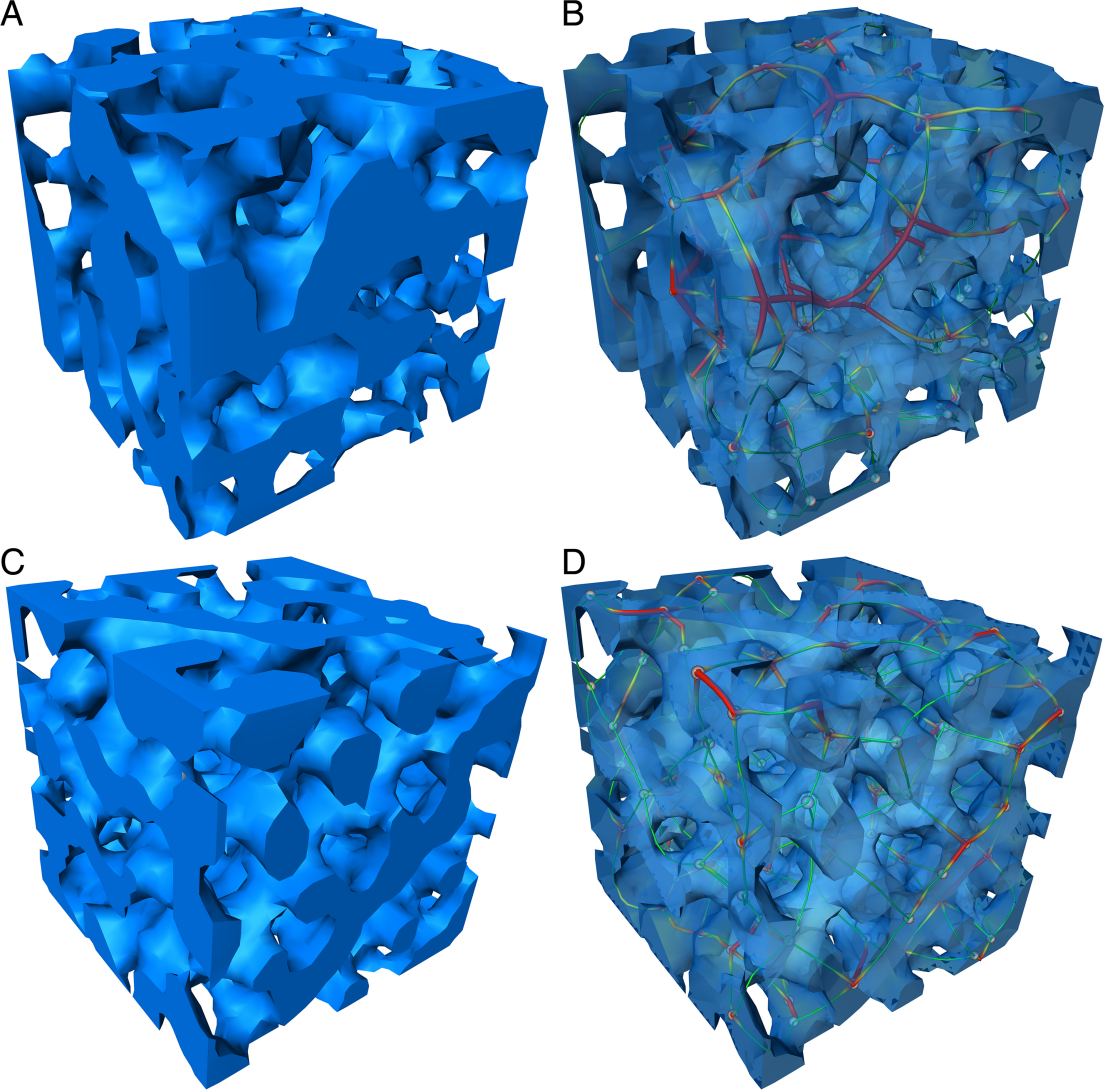
Stereographic rendering of the stereom. (A) Trabeculae at the plate’s center are unordered. (B) The visualization of thinned and color-coded trabeculae shows the sturdy trabeculae. (C) trabeculae at the plate’s margin are more ordered, and (D) the trabeculae are slenderer than those of the plate’s center.

The chord length (shortest distance between nodes) is on average 16.43 ± 5.8 μm (N = 1419), the curved length (true length of a trabecula) 17.56 ± 6.1 μm (N = 1419) (Fig 5A and 5B, Table 1). A Wilcoxon rank-sum test of chord length and curved length shows that the two length parameters are statistically not different at a significance level of α = 5% (p = 0.131, N = 600, iterations: 10000). The tortuosity of the segments is τ = 1.03 ± 0.0 (N = 1419) and is close to τ = 1. The segments show an average radius of 2.33 ± 1.2 μm (N = 1418), nodes show an average radius of 1.500 ± 0.0 μm (N = 712), t (Table 1). A Wilcoxon rank-sum test indicates that segment are on average thicker than the nodes based on a significance level of α = 5% (p < 0.001, N = 600, iterations: 10000). The average slenderness ratio is 15.24 ± 8.4 (N = 1418).

**Table 1:** Trabecular measurements and orientation.

position		median	mad	min	max	N
**center**	**segment chord length**	16.43	5.80	0.0	42.1	1418
	**segment curved length**	17.55	6.13	6.0	51.3	1418
	**segment tortuosity**	1.03	0.03	1.0	3.0	1415
	**segment radius**	2.33	1.21	1.5	7.6	1418
	**slenderness ratio**	15.24	8.44	2.70	51.96	1418
	**node radius**	1.50	0.00	1.5	9.0	711
	**orientation theta**	59.09	22.82	0.0	90.0	1418
	**orientation phi**	180.00	133.51	0.0	360.0	1418
**margin**	**segment chord length**	16.43	5.48	0.0	34.2	1559
	**segment curved length**	17.43	5.39	6.0	49.6	1559
	**segment tortuosity**	1.03	0.03	1.0	2.4	1559
	**segment radius**	2.22	0.79	1.5	5.3	1559
	**slenderness ratio**	16.14	7.76	2.77	52.57	1559
	**node radius**	1.50	0.00	1.5	5.0	774
	**orientation theta**	59.67	27.14	0.0	90.0	1559
	**orientation phi**	180.10	133.62	0.0	360.0	1559

mad = median absolute deviation, min = minimum value, max = maximum value, N = sample size.

#### Plate margin

The chord length (shortest distance between nodes) is on average 16.43 ± 5.5 μm (N = 1560), the curved length (true length of a trabecula) 17.43 ± 5.4 μm (N = 1560) (Fig 5B and 5C, Table 1). A Wilcoxon rank-sum test of chord length and curved length shows that the two length parameters are statistically not different at a significance level of α = 5% (p = 0.150, N = 600, iterations: 10000). The resulting tortuosity of the segments is τ = 1.03 ± 0.0 (N = 1560) and is close to τ = 1. The segments show an average radius of 1.50 ± 0.0 μm (N = 6543) (Table 1), nodes show an average radius of 1.50 ± 0.0 μm (N = 775). A Wilcoxon rank-sum test indicates that segment are on average thicker than the nodes based on a significance level of α = 5% (p < 0.001, N = 600, iterations: 10000). The average slenderness ratio is 16.14 ± 7.8 (N = 1559).

#### Comparison of the plate’s center and margin

The statistical analysis indicates that segments from both the plate’s center and the plate’s margin are similar in their length (p = 0.403, N = 600, iterations: 10000). The segment radius are similar between the two plate regions based on an significance level of α = 5% (p = 0.054, N = 600, iterations: 10000). The tortuosity of both plate regions statistically indistinguishable (p = 0.287, N = 600, iterations: 10000), as the node radius is (p = 0.375, N = 600, iterations: 10000). The slenderness ratios of the plate’s center and the plate’s margin are statistically not different (p = 0.303, N = 600, iterations: 10000).

### Inter-trabecular angle (ITA) and node configuration

#### Plate center

**Fig 6:**
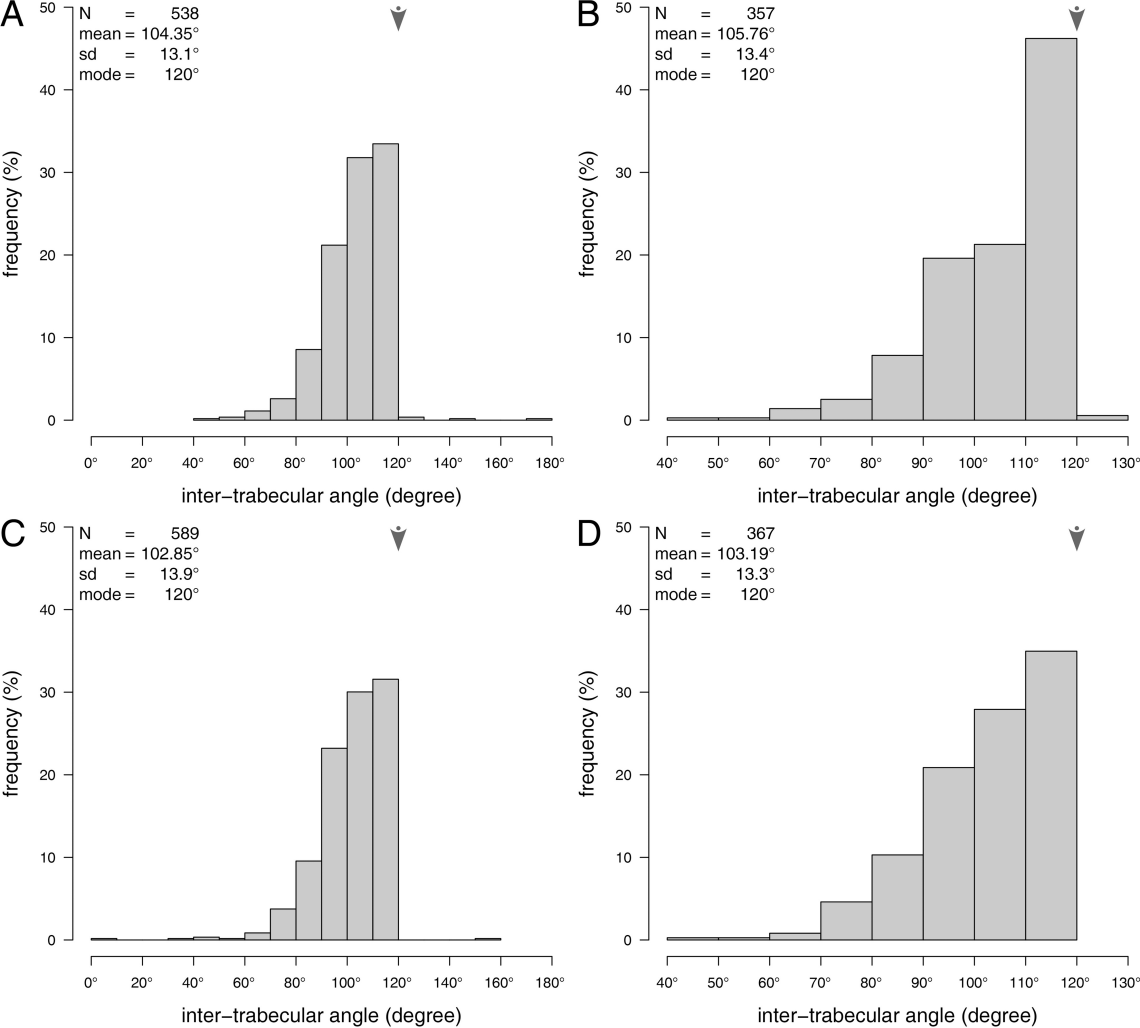
Histograms of the inter-trabecular angles. (A) All inter trabecular angles of the plate centers. (B) Inter trabecular angles of the plate centers, where 3 segments intersect in a single common node. (C) All inter trabecular angles of the plate margins. (D) Inter trabecular angles of the plate margins, where 3 segments intersect in a common node. N = sample size, med. = median, mad. = median absolute deviation, mode is indicated by arrows.

The analysis of the plates’ centers (Fig 5A and 5B) involved 1419 segments, which intersected in 538 nodes. In 1.3% of the 538 nodes, two segments intersected in one common node (Table 2), in 66.4%, three segments intersected in one common node, in 21.4%, four segments intersected in one node, in 6.5% five segments intersected in one node, in 3.9% six segments intersected in one node, and in 0.6% seven or more segments intersected in one node. The average inter-trabecular angle is ITA = 104.35 ± 13.1° (N = 538) with a mode of 120° (Fig 6A). The majority of intersection nodes involve three segments. In the cases where three segments share one common node, the ITA is 105.76 ± 13.4° (N = 357) with a mode of 120° (Fig 6B). In the cases where more than three segments intersect in one common node, the ITA decreases compared to 2N – 4N (Table 2).

**Table 2:** Inter-trabecular angles and node-configuration.

	ITA	total	2N	3N	4N	5N	6N	7N	8N	9N
**center**	**N**	538	7	357	115	35	21	2	n/a	1
	**fraction (%)**	100.00	1.30	66.40	21.38	6.51	3.90	0.37	n/a	0.19
	**mean (deg)**	104.35	117.19	105.76	102.40	99.68	95.88	95.27	n/a	99.68
	**sd (deg)**	13.1	30.2	13.4	10.2	10.7	9.6	4.7	n/a	n/a
	**mode (deg)**	120.00	146.87	120.00	98.91	94.96	85.03	91.96	n/a	n/a
**margin**	**N**	589	5	369	149	38	20	4	1	n/a
	**fraction (%)**	100.00	0.93	68.59	27.70	7.06	3.72	0.74	0.19	n/a
	**mean (deg)**	102.85	117.04	103.19	102.19	102.89	99.38	92.91	113.76	n/a
	**sd (deg)**	13.9	20.1	13.3	15.2	12.4	15.3	5.7	n/a	n/a
	**mode (deg)**	120.00	150.17	120.00	100.12	99.78	100.11	100.53	n/a	n/a

ITA = inter-trabecular angle, N = sample size, deg = degree, sd = standard deviation.

#### Plate margin

The analysis of the plates’ margins (Fig 5B and 5C) involved 1559 segments, these segments intersected in 589 intersection nodes. In 0.9% of the 589 nodes, two segments intersected in one common node, in 68.6%, three segments intersected in one node, in 27.7%, four segments intersected in one node, in 7.0% five segments intersected in one node, in 3.7% six segments intersected in one node, and in 0.9% seven or more segments intersected in one node (Table 2). The average inter-trabecular angle is ITA = 102.85 ± 13.9° (N = 589) with a mode of 120° (Fig 6C). The majority of intersection nodes involve three segments. In the cases where three segments share one common node, the ITA is 103.19 ± 13.3° (N = 369) with a mode of 120° (Fig 6D). In the cases where more than three segments intersect in one common node, the ITA decreases compared to 2N – 4N (Table 2).

#### Comparison of the plate’s center and margin

The inter-trabecular angles of the plate’s center and those of the plates margin are statistically not different as shown by the Wilcoxon rank sum comparison (p = 0.313, N = 600, iterations: 10000). The inter-trabecular angles between segments, where three segments intersect in one common node is likewise similar (p = 0.060, N = 600, iterations: 10000).

### Trabecular theta orientation

#### Plate center

**Fig 7:**
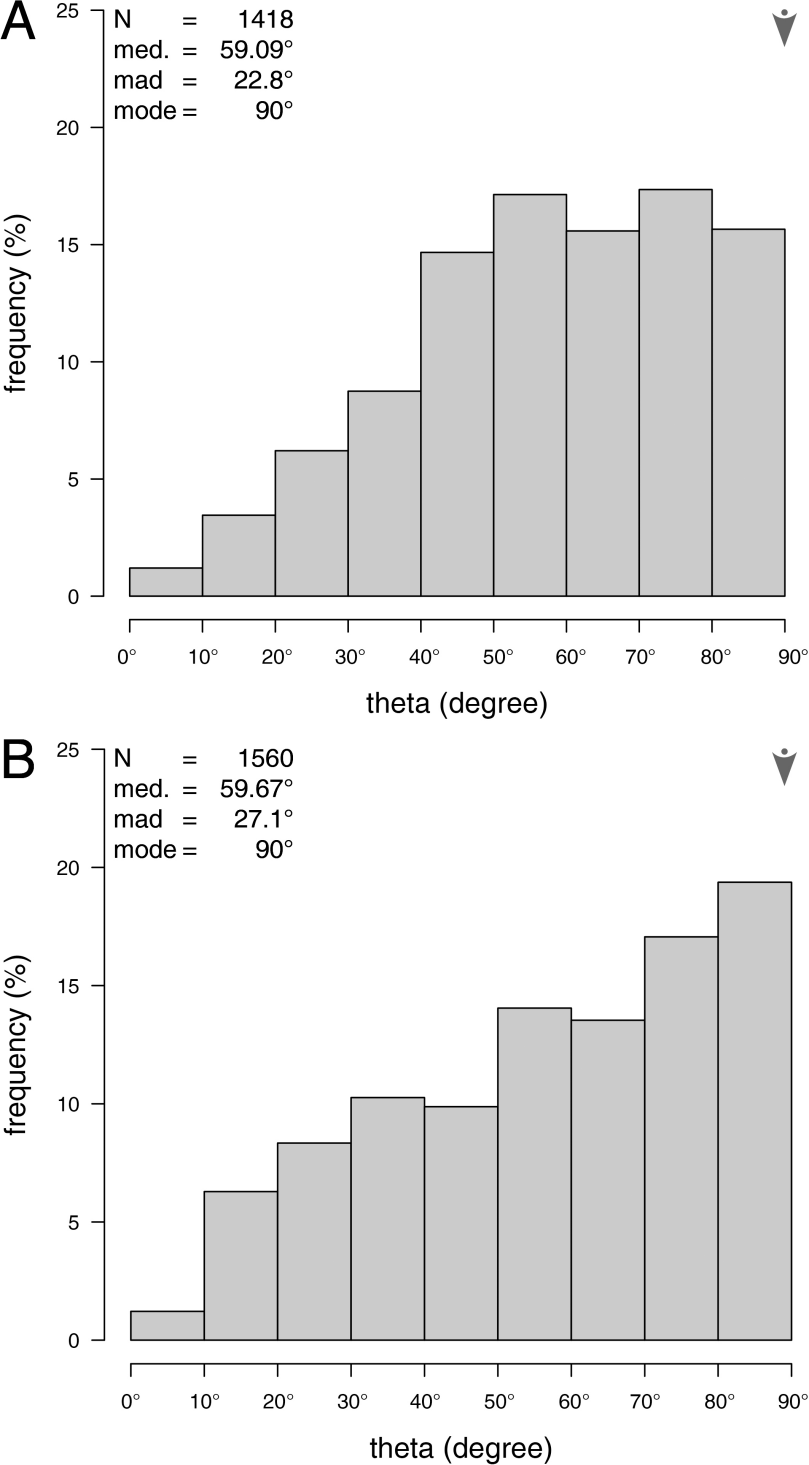
Histograms of the trabecular orientation with respect to the plate’s horizontal plane. (A) Trabecular distribution at the plate’s center. (B) Trabecular distribution at the plate’s margin. N = sample size, med. = median, mad. = median absolute deviation, mode is indicated by arrows.

The angle theta between the segments and the z-axis (axis perpendicular to the plate’s surface) is on average θ = 59.09 ± 22.8° (N = 1418, Fig 7A). A Shapiro-Wilk test for normality reveals that the distribution of theta is statistically different from a Gaussian distribution at a significance level of α = 5% (W = 0.972, p < 0.001, N = 1418). The distribution of theta is left skewed and flattened compared to a Gaussian distribution (skewness = -0.369, kurtosis = 2.480, N = 1419) (Fig 7A).

#### Plate margin

The angle theta between the segments and the z-axis (axis perpendicular to the plate’s surface) is on average θ = 59.67 ± 27.1° (N = 1560, Fig 7B). A Shapiro-Wilk test for normality reveals that the distribution of theta is statistically different from a Gaussian distribution at a significance level of α = 5% (W = 0.954, p < 0.001, N = 1560). (Fig 7B). The distribution of theta is left skewed and flattened compared to a Gaussian distribution (skewness = -0.351, kurtosis = 2.105, N = 1560).

#### Comparison of the plate’s center and margin

The trabecular orientation in relation to the z-axis (axis perpendicular to the plate’s horizontal plane) is similar at the plate’s center and the plate’s margin (p = 0.502, N = 600, iterations: 10000).

### Trabecular phi orientation

#### Plate center

**Fig 8:**
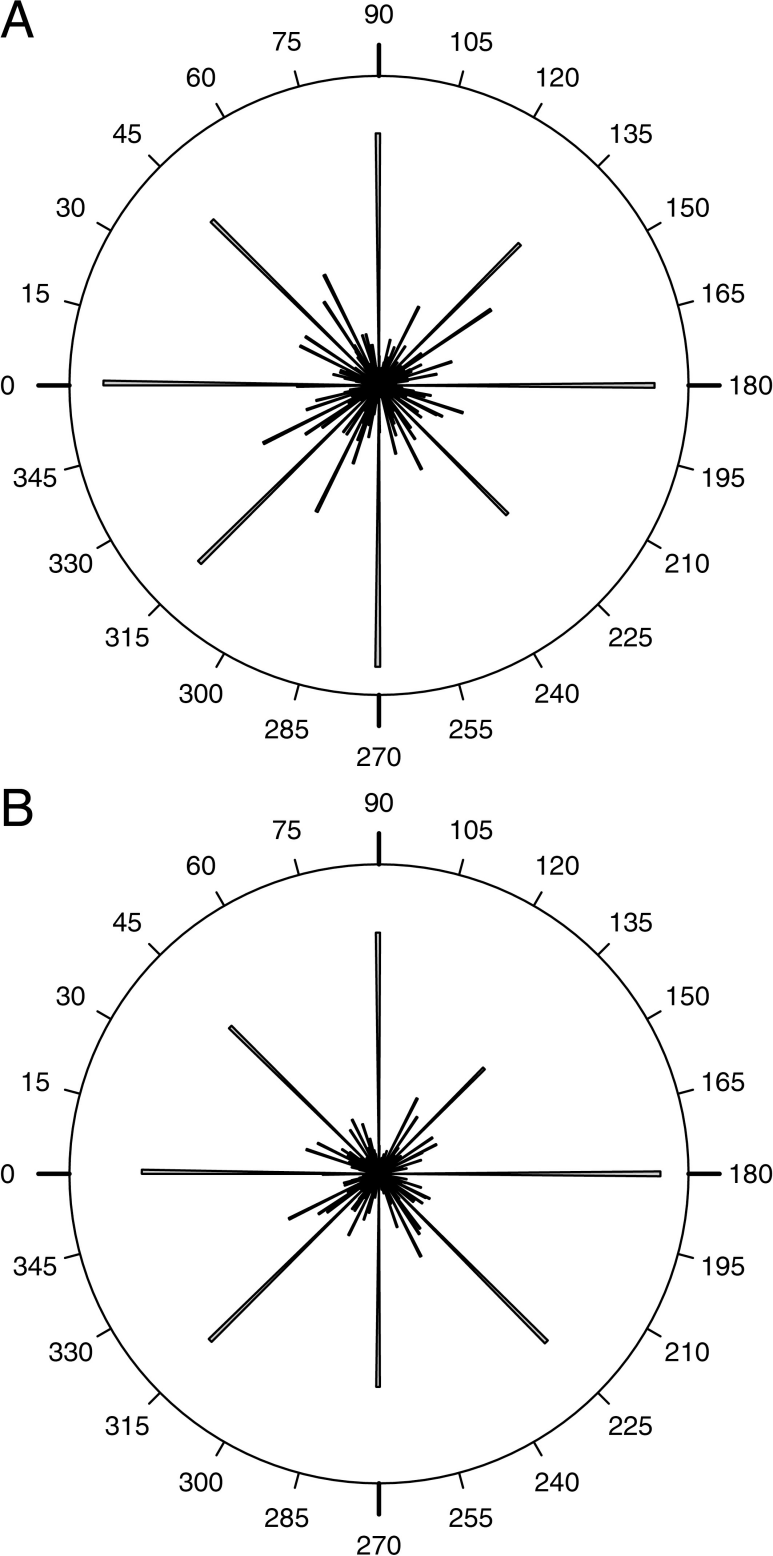
Rose diagrams of the trabecular orientation within the plate’s horizontal plane. (A) Phi distribution at the plate’s center. (B) Phi distribution at the plate’s margin. Both diagrams indicate that the trabeculae are directional aligned within the plate’s horizontal plane.

The trabecular orientation within the plate’s horizontal plane does not follow a uniform distribution (χ^2^(36) = 152.91, p < 0.001, N = 1418). The respective rose diagram indicates that trabeculae are directional orientated within the plate’s horizontal plane (Fig 8A).

#### Plate margin

The trabecular orientation within the plate’s horizontal plane does not follow a uniform distribution (χ^2^(36) = 303.13, p < 0.001, N = 1559). The respective rose diagram indicates that trabeculae are directional orientated within the plate’s horizontal plane (Fig 8B).

#### Comparison of the plate’s center and margin

The trabecular orientation within the plate’s horizontal plane is similar in both plate areas (p = 0.478, N = 600, iterations: 10000).

## Discussion

### Length and radius

The trabeculae follow a direct course between two nodes, demonstrated by tortuosity values close to τ = 1. The chord length and curved length are additionally statistically indistinguishable in and between both the plate’s center and margin (Table 1). The trabecular length at the plate’s center and at the plate’s margin, as well as the average segment radius are similar. The aspect ratio of trabecular length and trabecular radius indicates that buckling is not a structurally critical parameter in the two plate regions.

### Inter-trabecular angle (ITA) and node configuration

The inter-trabecular angles of the plate’s center and the plate’s margin are similar, with the overall ITA slightly lower than the ITA for three segments per node (3N). Although the average ITA at the plate’s center is 105.76° and 103.19° at the plate’s margin, the majority of ITA are in both regions at 120° (Fig 6B and 6D) indicating that the triangular geometrical configuration with a maximum segment is structurally advantageous for multi-directional stress handling. In the 3N configuration, more 120° angles are present on the plate’s center than in the plate’s margin (Fig 6B and 6D) indicating that the center is more likely adapted to multi-directional stress. This result can be explained by the load-distribution in the echinoid skeleton (Grun and Nebelsick 2018), where loads applied to a plate are distributed via the galleried stereom to adjoining plates.

**Fig 9:**
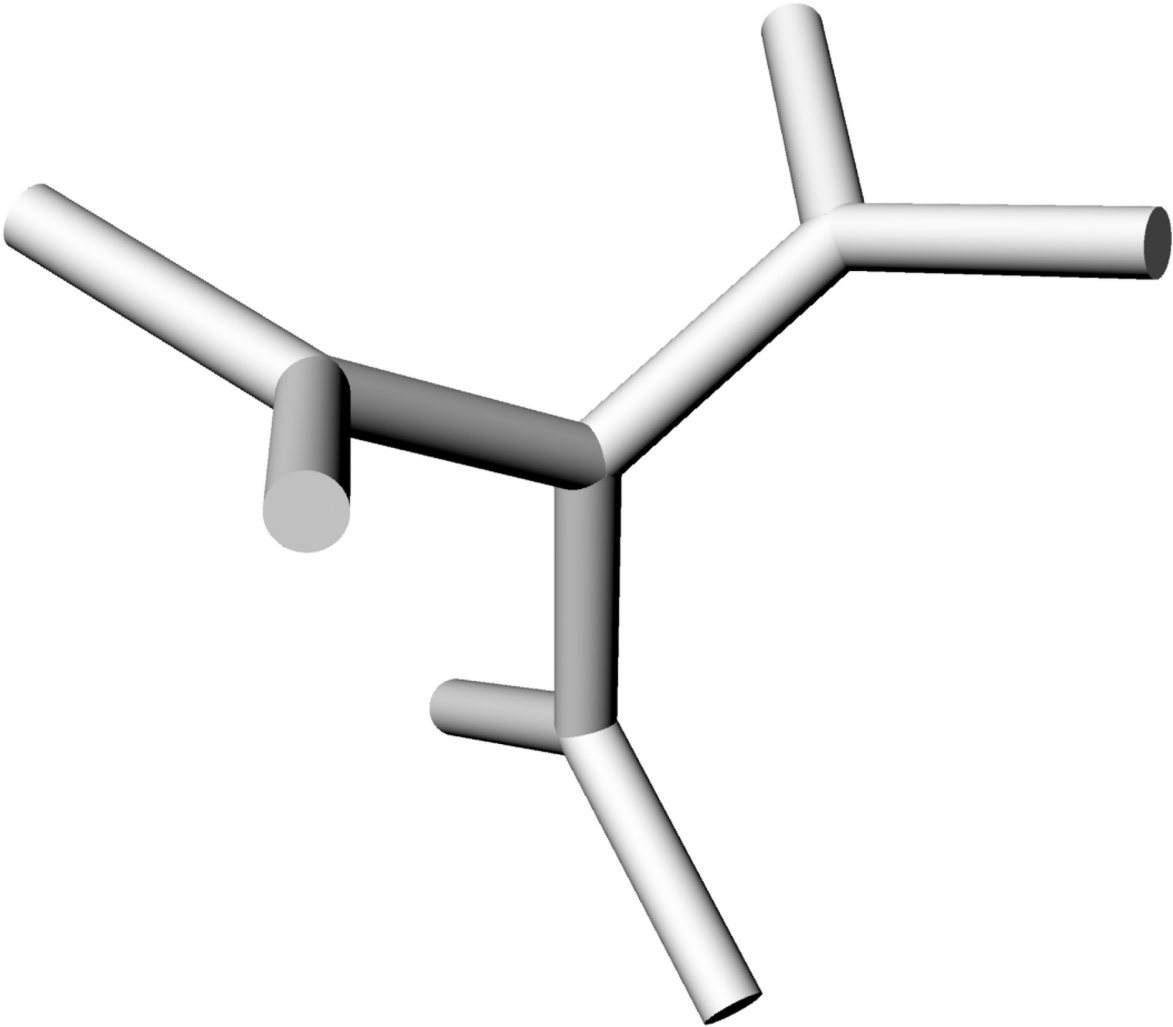
Lattice-system. Built-up from multiple 3N members. All inter-trabecular angles possess an offset of 120 degrees to one another.

Reznikov et al. (2016) demonstrated that the ideal ITA of a 3N configuration is 120°, as this angular motif spans the longest space. This 3N and 120° motif results in a planar and triangular geometric element [26]. In nodes where four segments share a common node (4N), the geometric motive is tetrahedral with a maximum segment offset of 109.5° [26]. Results show that the planar 120° triangular motifs account for around two thirds of the node configuration. In terms of its structural performance, the planar triangular geometry can be used to counter multi-dimensional loadings when arranged in an angular off-set (Fig 9).

### Trabecular theta orientation

Trabeculae of both the plate’s center and the plate’s margin show a similar distribution pattern of theta, which ranges between 0 and 90 degrees with the z-axis (Fig 4). In both plate areas, the mean angle formed by the trabeculae and the z-axis is around 60 degrees with a mode of 90 degrees (Fig 7). This distribution indicates that trabeculae are orientated in all spatial directions, capable to meet multi-directional stress [3, 26]. The most frequent theta for both the plate’s center and the plate’s margin is found at 90 degrees, which is parallel to the plates surface. The distribution of theta in the plate’s margin is slightly more left skewed than the distribution of theta in the plate’s center, which indicates a slightly, yet statistically insignificant higher frequency of 90 degree trabeculae (Fig 7). The orientation of the trabeculae within the plate is in contrast to general descriptions of trabecular orientations within echinoid plates, where the center is usually described as unordered labyrinthic stereom, whereas the marginal areas consist of the highly ordered galleried stereom [3].

The differentiation between labyrinthic and galleried stereom has been discussed with respect to their function: the labyrinthic stereom with its unordered trabeculae is considered to absorb loads from multiple directions, whereas the ordered galleried stereom can distribute loads from its point of origin along the echinoid’s skeleton [3, 10]. Interestingly, μCT sections [35] indicate that there is a visual separation between the center and the margin of a plate in *Echinocyamus pusillus*. The discrepancy between the statistical similarity of the trabeculae orientation in the plate’s center and margin (Fig 7) and the visual separation between both areas is interpreted to be based on two parameters: first, trabeculae at the plate’s center are assumed to counter loads from multiple directions. These loads need to be deflected from vertical loads to horizontal loads in order to be distributed by the marginal areas to other plates. Second, loads are applied to the entire surface of the echinoid, including both the plate’s center and the plate’s margin. Therefore, the margins also have to counter loads from above. Although the main direction of the primary trabeculae at the plate’s margins show a distinctive direction, other trabeculae vary in orientation to counter loads from multiple directions respectively to the plate’s center. Additionally, the separation of the plate’s margin and the plate’s center has always been analyzed from physical [3] or virtual [16] sections of a echinoid’s plate. This pioneering examination indicates that the spatial trabecular orientation of plates of the clypeasteroid *Echinocyamus pusillus* is similar within a plate.

### Trabecular phi orientation

The trabecular orientation within the plate’s horizontal plane in both the plate’s center and the plate’s margin are highly directed indicated by the angular distribution (Fig 8) and supported by statistical tests. The directional alignment of the trabecular system is indicative for a directional load transfer system. Applied loads are here interpreted to impinge on the echinoid’s shell surface, where the loads can be supported by the trabeculae in theta direction. The loads are then transferred into lateral thrust [36]. The directional trabeculae in phi direction can distribute this stresses laterally onto neighboring plates. The resulting stress is thereby dispensed which leads to a lower chance of structural failure.

## Conclusions

The echinoid’s trabecular system is characterized by the eight descriptors of (1) trabecular length, (2) trabecular tortuosity, (3) trabecular radius, (4) trabecular slenderness ratio, (5) inter-trabecular angle, (6) node configuration, (7) theta orientation, and (8) phi orientation. Single trabeculae are short, stocky and possess very little tortuosity. The minor slenderness ratio in echinoids indicate that their trabecular system is able to resist higher loads before the critical Euler buckling is reached. The majority of trabecular intersections follow the 3N configuration, where three trabeculae intersect in one common node. The most abundant ITA is thereby at 120°. The resulting triangular and planar geometry can, when combined, form a three-dimensional meshwork able to counter multi-dimensional stress. The trabecular orientation in z-direction indicates that the plate is capable of handling loads along the entire surface. The trabecular orientation within the plate’s horizontal plane is directional, enabling the plate to distribute loads to neighboring plates.
